# Over-Expression and Prognostic Significance of HHLA2, a New Immune Checkpoint Molecule, in Human Clear Cell Renal Cell Carcinoma

**DOI:** 10.3389/fcell.2020.00280

**Published:** 2020-05-19

**Authors:** Zhen Zhang, Jinyan Liu, Chaoqi Zhang, Feng Li, Lifeng Li, Dan Wang, Damini Chand, Fangxia Guan, Xingxing Zang, Yi Zhang

**Affiliations:** ^1^Biotherapy Center, The First Affiliated Hospital of Zhengzhou University, Zhengzhou, China; ^2^Department of Thoracic Surgery, National Cancer Center/Cancer Hospital, Chinese Academy of Medical Sciences and Peking Union Medical College, Beijing, China; ^3^Department of Microbiology and Immunology, Albert Einstein College of Medicine, Bronx, NY, United States; ^4^School of Life Sciences, Zhengzhou University, Zhengzhou, China; ^5^Cancer Center, The First Affiliated Hospital of Zhengzhou University, Zhengzhou, China; ^6^Key Laboratory for Tumor Immunology and Biotherapy of Henan Province, Zhengzhou, China

**Keywords:** KIRC, HHLA2, CD8^+^ T cells, TCGA, tissue microarrays

## Abstract

HHLA2, a newly identified B7 family member, regulates T cell functions. However, the expression and prognostic value of HHLA2 in solid tumors is ill defined. This study aimed to reveal the expression landscape of HHLA2 in various solid tumors, and to evaluate its prognostic value in kidney clear cell carcinoma (KIRC). Using The Cancer Genome Atlas (TCGA) database, we investigated the expression pattern of HHLA2 across 22 types of cancer. HHLA2 and CD8 protein expression was determined via immunohistochemistry (IHC). KIRC-specific findings were further analyzed with R software and the prognostic value was validated on tissue microarrays. HHLA2 was widely expressed in cancers at both the mRNA and protein levels. Among all tested tumors, KIRC showed the highest transcript level of HHLA2, and HHLA2 levels were significantly higher in tumor tissues than in matched normal samples, as evidenced by both TCGA and IHC data. HHLA2 was also positively correlated with survival rates in KIRC based on TCGA and clinical data. Receiver operating characteristic curves data showed the prognostic value of HHLA2 for patients with KIRC in TCGA. Moreover, HHLA2 was positively correlated with immune-related genes, while HHLA2 and CD8 expression exhibited a consistent trend in KIRC tumor samples. In conclusion, HHLA2 is highly expressed in KIRC and predicts a favorable survival outcome, highlighting that it may work as a potential target for KIRC therapy.

## Introduction

Renal cell carcinoma (RCC) accounts for 3% of adult malignant tumors and ranks as the most lethal of all urologic cancers (Ferlay et al., 2015). Nearly 95% of RCC cases are clear cell KIRC, KIRP, and KICH (Shuch et al., 2015). Among these three types, KIRC shows strong resistance when treated with traditional therapies, including chemotherapy and radiotherapy, with a < 20% 2-year survival rate for metastatic patients (Chen et al., 2016; Han et al., 2017). During the last decade, immunotherapy has attracted attention due to the important role of the immune system in cancer (Schumacher and Schreiber, 2015; Ribas and Wolchok, 2018). It has been clearly demonstrated that once an antigen is presented through the MHC, a simultaneous signal is required to determine the type of T cell response. A costimulatory signal induces T cell activation while a coinhibitory signal results in T cell inhibition (Shi et al., 2019). In one mechanism of tumor evasion from the immune system, tumor cells express coinhibitory ligands that result in T cell exhaustion and blunt the immune response (Chen et al., 2018; Zhang et al., 2020). By targeting this phenomenon, immune checkpoint blockade therapy (ICBT), can unleash the breaks in the immune system and induce long-lasting responses (Hato et al., 2014; Marin-Acevedo et al., 2018a). With these advances in immunotherapy, patients with KIRC have been treated with PD-1 inhibitors; however, the limited expression of PD-Ll limits application of this therapy in the clinic (Shi et al., 2019). Thus, identifying a more suitable target in KIRC to improve immunotherapy efficacy is necessary.

The B7 and CD28 family have attracted increasing attention for their important roles in determining T cell fate. Inhibiting coinhibitory checkpoints using ICBT has been regarded as a promising method for controlling tumors, and many studies have demonstrated its efficacy (Li et al., 2018; Marin-Acevedo et al., 2018b). The most studied are PD-1 and CTLA-4, which have been approved by the FDA for the treatment of blood tumors (Hirano et al., 2005; Yu et al., 2016; Chau, 2017). Although this approach has provided encouraging results, the clinical responses are far from perfect for patients with solid cancers (Pardoll, 2012; Topalian et al., 2012; Abril-Rodriguez and Ribas, 2017); the underlying mechanisms resulting in treatment failure are complicated. Thus, exploring new targets may help increase the efficacy of ICBT. HHLA2 (B7H7/B7-H5/B7y), a newly defined B7 family member (Zhao et al., 2013), is a co-inhibitory molecule expressed in multiple cancers, including lung, breast and pancreatic cancers as well as melanoma, and osteosarcoma and shows limited expression in normal tissues. While HHLA2 expression is associated with worse survival in patients with osteosarcoma (Koirala et al., 2016), its expression and significance of HHLA2 is ill defined in other types of solid tumors (Janakiram et al., 2015b). Therefore, in this study, we aimed to reveal the expression pattern and potential prognostic value of HHLA2 in various solid tumors.

## Materials and Methods

### The Cancer Genome Atlas (TCGA) Database

HHLA2 transcriptome data from 22 types of solid tumors in TCGA dataset were obtained from the website of the Cancer Genomics Browser of the University of California Santa Cruz^1^. The following tumor types were selected: KIRC (*n* = 534), READ (*n* = 95), KIRP (*n* = 291), COAD (*n* = 288), PAAD (*n* = 179), LUAD (*n* = 517), ESCA (*n* = 185), LUSC (*n* = 502), OV (*n* = 308), PRAD (*n* = 498), LGG (*n* = 530), THYM (*n* = 120), HNSC (*n* = 522), CESC (*n* = 305), PCPG (*n* = 184), LIHC (*n* = 373), KICH (*n* = 66), GBM (*n* = 167), BLCA (*n* = 407), SKCM (*n* = 473), SARC (*n* = 263), BRCA (*n* = 1104). We also retrieved KIRC normal sample (*n* = 72) data from TCGA. Only primary patients were enrolled in this study while recurrent ones were excluded.

### Gene Expression Omnibus (GEO) Datasets

Normalized data of a previous Affymetrix HG-U133A 2.0 gene expression array that compared gene expression in KIRC tumors and matched adjacent normal tissues was downloaded from the GEO^2^. Specifically, 101 and 72 pairs of normal and matched cancer samples were obtained from GSE40435 and GSE53757, respectively. While 63 cases of normal samples and 67 cases of cancer samples were obtained from GSE46699. Network Analyst software was used to re-analyze the data.

### DNA Methylation Analysis

We collected DNA methylation datasets from 319 KIRC cases in TCGA program. Methylation measurements were performed using the Illumina Human Methylation 450 platform (Illumina, San Diego, CA, United States). HHLA2 gene expression values from KIRC tumor tissues were also extracted. Pearson’s product-moment correlation between HHLA2 gene expression levels and methylation of its CpG islands was evaluated. Data analysis was performed using R software^3^. Data analysis was completed by using MEXPRESS^4^.

### Patients and Samples

All paraffin-embedded tumor tissue specimens (*n* = 250) were collected from patients with KIRC, who underwent surgery at the First Affiliated Hospital of Zhengzhou University. Normal and tumor tissue microarrays (TMAs) were purchased from Shang Hai Outdo Biotech for the analysis of HHLA2 expression in human tissues. The diameter of the tissue chip was 1 mm. The types of tumors in TMAs were listed as follows: KIRC, STAD, COAD, LUAD, BLCA, BRCA, ESCA, PAAD, UCEC, READ, THCA, and CESC. TMA construction has been previously described in detail (Nocito et al., 2001). This study was approved by the Ethics Committee of the First Affiliated Hospital of Zhengzhou, Henan, China.

### Immunohistochemistry (IHC) Staining

To examine HHLA2 expression in tumors and matched normal tissue, samples from cancer patients were obtained from the First Affiliated Hospital of Zhengzhou University. Tumor tissues were incubated in 4% paraformaldehyde (PFA) overnight, then embedded in paraffin, and sectioned at a thickness of 4 μm. For staining, the slides were deparaffinized and rehydrated, followed by antigen retrieval. The sections were then blocked with 5% BSA in PBS and incubated with anti-HHLA2 mAb (2 μg/mL, 1:500; clone 566.1, IgG1) (Cheng et al., 2017) or anti-CD8 (1:200; ab93278; Abcam, Cambridge, United Kingdom) monoclonal antibodies at 4°C overnight. The next day signal amplification was performed using an ABC HRP Kit (Zhongshanjinqiao Biotechnology, Beijing, China) and the samples were counter-stained with hematoxylin. Following dehydration with a graded ethanol series and clearing with xylene, the sections were imaged using a microscope (Leica, Wetzlar, Germany). Non-immune immunoglobulin G (IgG) was used as negative control. To analyze HHLA2 and CD8 expression, 5 to 10 fields were analyzed per patient sample. Marker density was scored independently by two investigators as follows: 0, negative; 1, weak; 2, moderate; or 3, strong. When tumor samples were tabulated, those with moderate and strong staining for HHLA2 or CD8 were considered the high expression group, while those with negative and weak expression were considered the low expression group.

### Gene Ontology (GO) Analysis

Immune-correlated genes were shared by TCGA dataset. Using DAVID Bioinformatics Resources 6.8^5^, function annotation was performed and the optimal related genes (top 600 genes ranked by Pearson |R|) were identified for analysis. Then, the groups on the network to the cluster distribution over the terms was visualized using the Cytoscape plugin (ClueGO plugin).

### Statistical Analysis

Statistical analysis was mainly performed by using R software^3^. All data are presented as mean ± SD. The association between HHLA2 expression and clinical parameters was analyzed using the χ^2^ test (age, sex, tumor grade, and stage) or Student’s *t*-test [overall survival, (OS)]. Univariate and multivariate analyses were performed using Cox proportional hazard model. Statistical analysis was also performed using the GraphPad Prism v6.0 (GraphPad Software, La Jolla, CA, United States). For the analysis of TCGA data, continuous variables were dichotomized for OS before running a log-rank test by employing optimal cutoff values. Comparison of the Kaplan–Meier survival curves between HHLA2 or CD8 expression high and low expression groups was performed using log-rank test. *P*-values < 0.05 were considered statistically significant.

## Results

### The Expression Landscape of HHLA2 in Different Types of Solid Tumors

**FIGURE 1:**
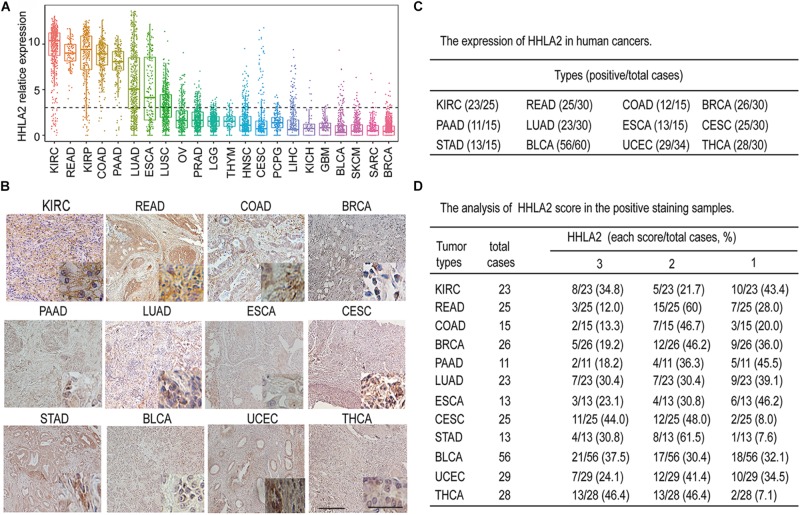
The expression landscape of HHLA2 in different types of solid tumors. **(A)** mRNA gene expression of HHLA2 in 22 tumor types was analyzed by using the mRNA-sequencing data from the TCGA database. **(B)** Representative IHC of HHLA2 expression in 12 types of tumor tissues. Full-sized images were acquired at 10 × and zoomed-in images were originally acquired at 40 ×. **(C)** HHLA2 protein expression in human cancers detected by immunohistochemistry via tissue microarrays; positive cases were analyzed. **(D)** The HHLA2 score was evaluated in each sample based on positive staining and intensity. A summary of HHLA2 score in human cancers was listed.

Accumulating evidence has shown that the B7 family of ligands provides coinhibitory or costimulatory signals that determine T cell fate (Schildberg et al., 2016). As the most recently discovered ligand of the B7 family, HHLA2 has attracted increasing attention (Janakiram et al., 2017). However, the expression pattern of HHLA2 in solid tumors is not well-defined. To comprehensively analyze the expression pattern of HHLA2 expression in solid tumors, we analyzed its transcriptome expression from TCGA data. As shown in Figure 1A, HHLA2 mRNA was widely expressed in 22 types of solid tumors and showed had expression in seven tumors including KIRC, READ, KIRP, COAD, PAAD, LUAD, ESCA according to the mean value. To further validate these findings, we performed IHC using TMAs. Consistent with TCGA data, HHLA2 was widely expressed in 12 types of solid tumors in protein level, including KIRC, READ, COAD, BRCA, PAAD, LUAD, ESCA, CESC, STAD, BLCA, UCEC, and THCA (Figure 1B). Furthermore, HHLA2 showed highly positive expression (Figure 1C) and positive intensity (Figure 1D). Taken together, these results demonstrate that HHLA2 was widely expressed in human solid cancers, and that it had high positive rate and expression in KIRC.

### HHLA2 Is Highly Expressed in Tumor Tissues and Predicts Good Outcomes in KIRC Patients

**FIGURE 2:**
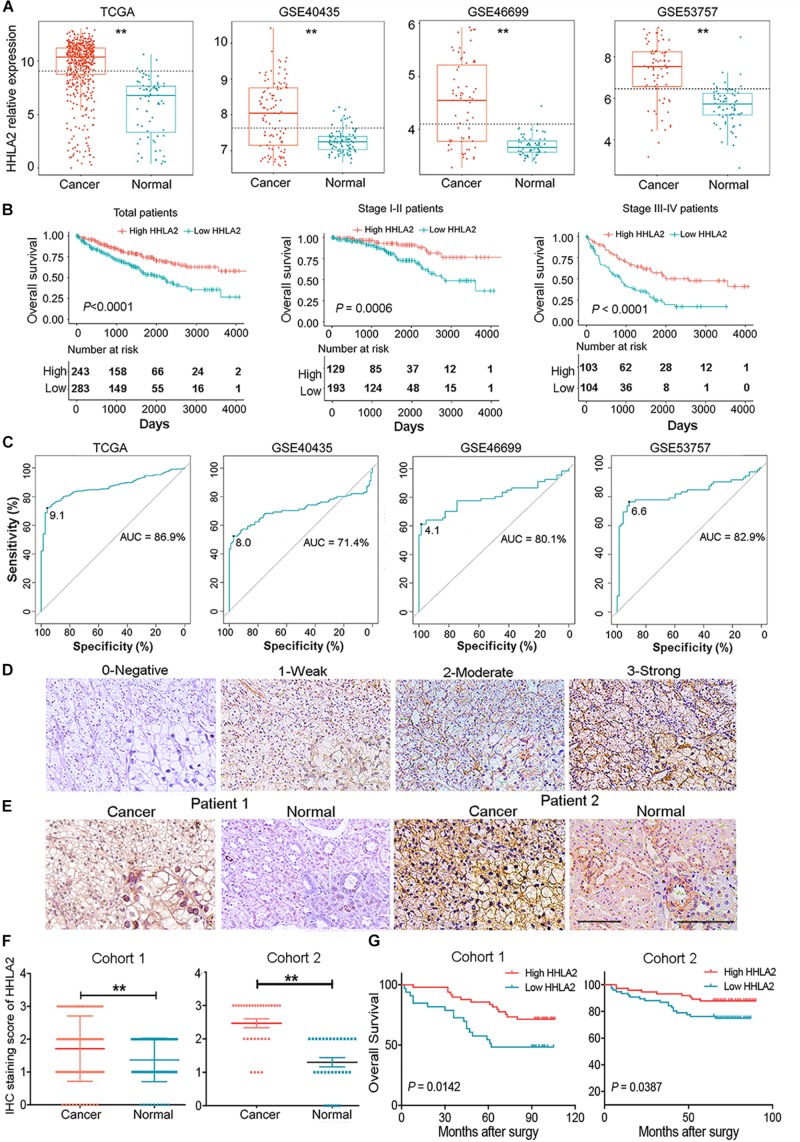
HHLA2 is highly expressed in tumor tissues and is a positive prognostic marker for patients with KIRC. **(A)** Compared with normal tissues, HHLA2 expression was higher in tumor tissues from patients with KIRC, based on TCGA and GEO datasets. **(B)** HHLA2 expression levels were positively correlated with the survival rates in KIRC from TCGA dataset. **(C)** The ROC curve of HHLA2 was analyzed by using the data from TCGA and GEO. **(D)** Evaluation of the HHLA2 score. Magnification, 10 and 20×. **(E)** Normal and tumor tissues from patients with KIRC were stained determine HHLA2 protein expression. **(F)** The HHLA2 score was significantly higher in the tumor tissues than that in normal samples from the KIRC patients (TMAs numbered Hkid-CRC180 Sur-01 was defined as cohort 1 and HkidE180 Su02 defined as cohort 2). **(G)** IHC staining showed that HHLA2 expression is positively correlated with survival time of patients with KIRC. **P < 0.01. Score 0, negative; 1, low expression; 2, moderate expression; and 3, high expression.

Given high positive rate and expression intensity of HHLA2 observed in KIRC samples, we next explored the significance of HHLA2 expression in patients with KIRC. First, we analyzed HHLA2 expression in KIRC tumors and matched normal tissues from TCGA dataset and found that HHLA2 expression is significantly higher expression in KIRC tumor tissues than in matched normal samples. Similar results were obtained with the GEO dataset (Figure 2A, all *P* < 0.01), indicating that HHLA2 is overexpressed in KIRC tumors. To investigate the potential significance of accumulated HHLA2, we divided the KIRC cohort (*n* = 531) into HHLA2-high and low expression groups and evaluated the prognostic value. Patients with high HHLA2 expression had a significantly longer survival time than those patients with low expression. When patients were divided into stage I–II and III–IV groups, similar results were obtained for both groups (Figure 2B, all *P* < 0.01). We further compared the clinical characteristics (including age, sex, clinical stage, and tumor grade) between HHLA2-high and low groups and observed statistically significant differences in tumor grade and survival; no significant differences were detected for other clinical features (Table 1). Furthermore, the univariate and multivariate Cox regression model using TCGA data revealed that age, tumor grade, clinical stage, and HHLA2 expression were independent prognostic factors for OS of patients with KIRC (Table 2). Evaluation of the correlation between tumor HHLA2 and clinicopathologic parameters in KIRC patients from two TAMs (*n* = 90 and *n* = 150) and 250 clinical samples revealed that tumor grade was correlated with HHLA2 expression in the validation 1 (*n* = 150) and 2 (*n* = 250) groups (Table 3). In addition, the area under the curve (AUC) of HHLA2 from TCGA and GEO data sets were 86.9, 71.4, 80.1, and 82.9%, respectively (Figure 2C), supporting HHLA2 as a prognostic marker in KIRC.

**TABLE 1:** Comparison of clinical characteristics between low HHLA2 group and high HHLA2 group in KIRC cohort.

**Variable**	**Case NO. (%)**	**HHLA2**		***P***
		**Low**	**High**	
Sample	531	266	265	
Age (year)				0.075
≤60	264	122	142	
>60	267	144	123	
Gender				0.192
Male	345	180	165	
Female	186	86	100	
Grade				0.001
G1	13	6	7	
G2	229	110	119	
G3	205	89	116	
G4	76	54	22	
Unknown	8	7	1	
Tumor stage				0.168
T1	271	137	134	
T2	69	35	34	
T3	180	85	95	
T4	11	9	2	
Clinical stage				0.888
I	265	134	131	
II	57	30	27	
III	123	58	65	
IV	84	43	41	
Unknown	2	1	1	
Distant metastases				0.341
Yes	107	58	49	
No	422	207	215	
Unknown	2	1	1	
Lymph node metastasis				0.629
Yes	291	143	148	
No	240	123	117	
Survival state				0.001
Alive	356	160	196	
Dead	175	106	69	
				

**TABLE 2:** Univariate and multivariate regression analyses for predicting overall survival in KIRC cohort.

**Variable**	**Univariate**		**Multivariate**	
	
	**HR (95% CI)**	***P***	**HR (95% CI)**	***P***
Age	1.8 (1.3–2.5)	<0.001	1.6 (1.2–2.2)	0.005
Gender	1.1 (0.8–1.5)	0.690	1.1 (0.8–1.5)	0.553
Laterality	0.7 (0.5–1.0)	0.028	0.9 (0.6–1.2)	0.407
Tumor grade	2.3 (1.8–2.8)	<0.001	1.4 (1.1–1.8)	0.003
Clinical stage	1.8 (1.6–2.1)	<0.001	1.8 (1.1–2.9)	0.013
Tumor stage	1.9 (1.6–2.2)	<0.001	0.8 (0.5–1.2)	0.288
Lymph node metastasis	0.9 (0.8–1.1)	0.257	0.9 (0.8–1.1)	0.225
Distant metastasis	4.3 (3.1–5.8)	<0.001	1.3 (0.7–2.6)	0.427
HHLA2 mRNA level	0.9 (0.8–0.9)	<0.001	0.8 (0.8–0.9)	<0.001

**TABLE 3:** Clinicopathologic characteristics of HHLA2 expression in KIRC patients from the discovery and validation groups.

**Parameter**	**Discovery cohort (*n* = 90)**			**Validation cohort 1 (*n* = 150)**			**Validation cohort 2 (*n* = 250)**		
			
	**HHLA2 Low**	**HHLA2 High**	***P***	**HHLA2 Low**	**HHLA2 High**	***P***	**HHLA2 Low**	**HHLA2 High**	***P***
Age^a^ (year)			0.800			0.899			0.776
<65	25	34		61	60		92	90	
≥65	14	17		15	14		33	35	
Gender^a^			0.966			0.977			0.241
Female	17	22		22	21		52	43	
Male	22	29		54	53		73	82	
Tumor grade^a^			0.448			<0.001			0.006
I	15	25		38	22		52	57	
II	17	21		19	43		40	53	
III	7	5		18	9		30	10	
IV	0	0		1	0		4	5	
Stage^a^			0.357			0.305			0.891
I	28	32		61	61		96	98	
II	7	11		7	9		16	18	
III	3	1		7	4		8	6	
IV	0	2		1	0		3	2	
Unknown	1	5		0	0		2	1	
Overall survival^b^ (months)	63.94	84.22	0.014	63.63	73.10	0.039	34.50	39.76	<0.001

*^*a*^χ^2^ test; ^*b*^Student’s *t*-test.*

We further examined protein expression of HHLA2 in TMAs and analyzed its correlation with patient prognosis. HHLA2 positive expression was defined as positive membranous and cytoplasmic staining (Figure 2D, staining scores of 0, 1, 2, or 3) as previous report (Cheng et al., 2017). HHLA2 showed significantly higher expression in tumor tissues than that of the matched normal samples in both cohort 1 (paired, *n* = 90) and cohort 2 (paired, *n* = 30) (Figures 2E,F). Kaplan–Meier curves was used to analyze the correlation between HHLA2 expression and OS, which showed that patients in the HHLA2-high group had longer survival rates than those of patients in the HHLA2-low group in both cohort 1 and 2 (Figure 2G), which was consistent with TCGA data. Together, these results demonstrated that HHLA2 was highly expressed in the tumor sites, and that increased expression is positively correlated with OS of patients with KIRC.

### Analysis of the Potential Genetic and Epigenetic Alterations Associated With HHLA2 Dysregulation

**FIGURE 3:**
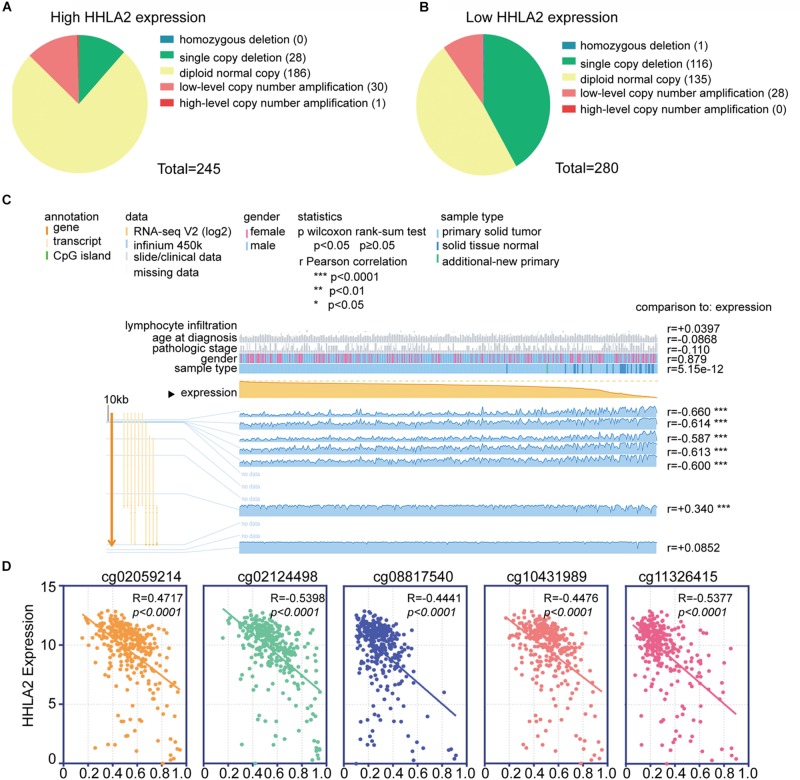
Analysis of the potential genetic and epigenetic alterations associated with HHLA2 dysregulation. **(A,B)** The CNAs in HHLA2-high and low expression groups. **(C)** Analysis of CpG island methylation and abnormal HHLA2 expression using TCGA dataset. **(D)** Correlation between HHLA2 expression and CpG island methylation was performed. ****P < 0.0001.

We next investigated the underlying mechanism underlying HHLA2 expression that induced abnormalities of HHLA2 in KIRC. Copy number alterations (CNAs) are an important mechanism of oncogene activation in cancer (Curtis et al., 2012). To reveal whether CNAs are responsible for the abnormal expression of HHLA2 in KIRC, we analyzed data from 415 cases with CNAs available in TCGA dataset. Different patterns of CNAs were observed in the HHLA2-high and low groups. Specifically, the proportion of the diploid normal copy was higher in the HHLA2-high group (75.9%, 186/245, Figure 3A) than in the HHLA2-low group (48.2%, 135/280, Figure 3B). The proportion of single copy deletions showed the opposite trend, with 41.4 and 11.4% in the HHLA2-low and high groups, respectively (Figures 3A,B). No differences were observed for homozygous deletion, low-level copy number amplification, and high-level copy number amplification between the two groups (Figures 3A,B).

Apart from CNAs, studies integrating DNA-methylation profiles and gene expression data have shown that methylation at different genomic regions is associated with gene expression levels (Diaz-Lagares et al., 2016; Chen G. et al., 2019). CpG DNA methylation is considered perhaps the most fundamental molecular phenomenon determining chromatin accessibility to the transcriptional machinery, thereby regulating gene expression (Chen G. et al., 2019). To investigate whether DNA methylation results in HHLA2 dysfunction, we examined status of 12 CpG sites of 319 KIRC cases in TCGA. Only seven CpG sites had associated data; among them, five CpG sites, including cg02059214, cg02124498, cg08817540, cg10431989, and cg11326415, showed a negative correlation with HHLA2 expression; the other two sites were positively correlated with HHLA2 (Figure 3C). We then employed the Pearson correlation coefficient and found that HHLA2 expression was negatively correlated with the above-mentioned five CpG sites (Figure 3D, all *P* < 0.01). Collectively, these data demonstrated the potential function of CNAs or DNA methylation in regulating the abnormal expression of HHLA2 in KIRC, highlighting the need for further investigation of the underlying mechanism.

### HHLA2 Is Positively Correlated With the Inflammatory Activities

**FIGURE 4:**
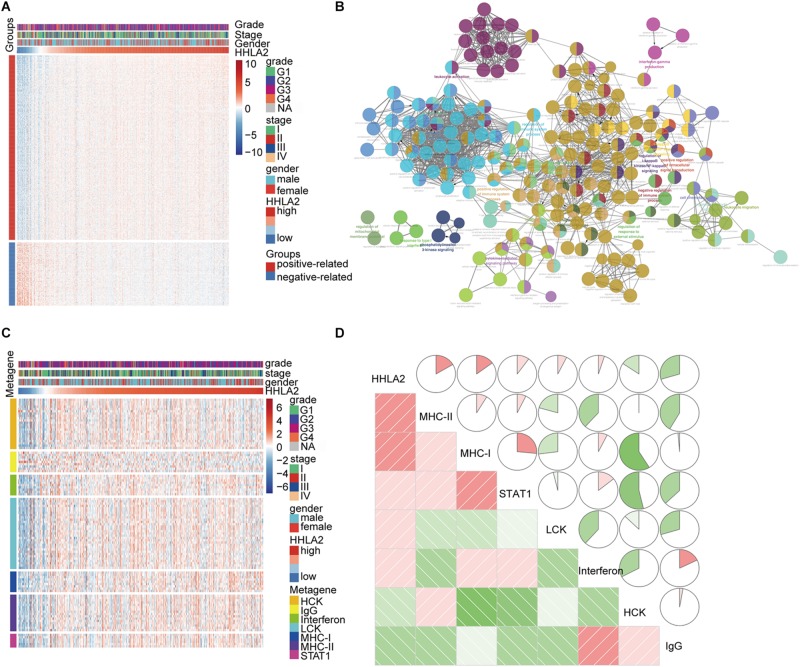
HHLA2 is positively correlated with an inflammatory gene expression profile. **(A)** HHLA2 correlation with inflammatory genes. **(B)** Gene ontology analysis of HHLA2 expression in KIRC. **(C)** HHLA2 correlation with immune responses from clusters derived from the TCGA data. **(D)** Correlation analysis between HHLA2 and immune-related genes using TCGA data.

Given that HHLA2 expression in KIRC is strongly associated with prognosis, we theorized that HHLA2 expression may be regulated by inflammatory responses, leading to enhanced survival rates. To identify the HHLA2 associated immune signature in KIRC, gene sets associated with the immune response^6^ were selected. We found 600 genes in TCGA dataset that were strongly correlated with HHLA2 expression (Pearson |R| > 0.5), and thus were eligible for further analysis. As most genes were positively correlated with HHLA2 expression (Figure 4A), we performed GO analysis to clarify their biofunctions. The results showed that these genes positively related to HHLA2 were significantly enriched in IFN-γ production, cell chemotaxis, and cytokine-mediated signaling pathway (Figure 4B). Thus, HHLA2 expression appears positively correlated with immune-related genes and immune responses in KIRC.

To gain a further understanding of HHLA2-related inflammatory activities, we chose seven clusters that were subsequently defined as metagenes from TCGA dataset. These genes represented different types of inflammatory and immune responses, and most clusters were positively associated with HHLA2 expression except for IgG, which was mainly associated with the activities of B lymphocytes (Figure 4C). To assess these cluster data, seven metagenes were generated using the gene sets variation analysis (GSVA) results of corresponding gene clusters. Then the correlogram was used to display relationships between variables, based on the Pearson *r* values between HHLA2 and the seven metagenes (Figure 4D). HHLA2 was positively correlated with MHC-II, MHC-I, STAT1 and LCK, but was negatively associated with IgG, consistent with what was observed in Figure 4A. Together, these results suggested that HHLA2 is positively correlated with inflammatory activities in KIRC.

### HHLA2 Is Not Simultaneously Expressed With Other B7-CD28 Family Members

**FIGURE 5:**
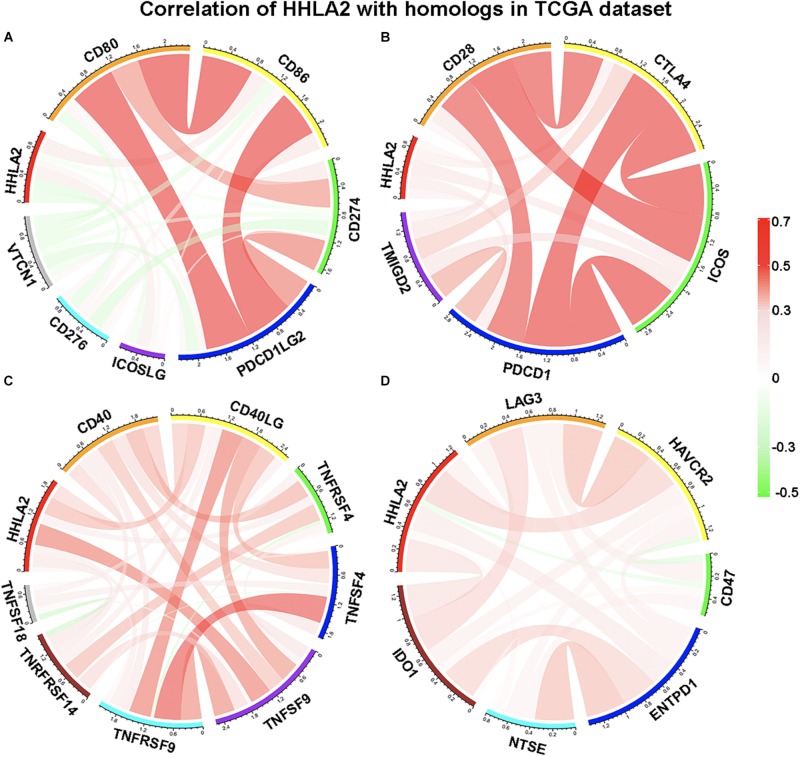
HHLA2 expression is not co-expressed with B7 or CD28 family. The B7 family **(A)**, CD28 family **(B)** and other immune check points **(C,D)** were used to analyze the correlation with HHLA2.

We next examined whether HHLA2 is co-expressed with other B7 family members, including CD80, CD86, CD274, CD276, ICOSLG, PDCD1LG2, and VTCN1. Pearson correlation coefficient analysis was performed with these factors in the TCGA database. Interestingly, we observed that HHLA2 showed no obvious correlation with CD80, CD86, CD274, CD276, ICOSLG, PDCD1LG2, or VTCN1 (Figure 5A), indicating that HHLA2 expression is not co-expressed with other B7 family members. Considering that B7 family members must bind to cognate CD28 family receptors expressed on T cells—e.g., CD80/CD86 binds to CD28—to regulate T cell function (Ville et al., 2015). Thus, we analyzed the correlation between HHLA2 expression with CD28 family, including CD28, CTLA-4, PDCD1, TMIGD2 and ICOS. Surprisingly, no obvious correlation was observed between HHLA2 and any CD28 family member (Figure 5B), suggesting that HHLA2 is not co-expressed with the CD28 family (Zhao et al., 2013).

It is well-known that activated immune response and inflammatory responses represent a good prognosis for patients with cancer. Tumor necrosis factor (TNF), a pleiotropic cytokine and a major mediator of apoptosis as well as inflammation and immunity, is a component of the immune response (Cervera-Carrascon et al., 2017). Accordingly, we analyzed the correlation between HHLA2 expression and the TNF family, including CD40, CD40L, TNFRSF4, TNFSF9, TNFRSF9, TNFSF18 and TNRFRSF14. A limited correlation was observed between HHLA2 and TNFSF9, while no clear correlation was observed with other family members (Figure 5C). As for other immune checkpoints such as LAG3, HAVCR2, CD47, ENTPD1, NTSE and IDO1, we found that none were strongly correlated with HHLA2 (Figure 5D). These results indicated that HHLA2 is not co-expressed with other B7 or CD28 family members, suggesting that it may work as an independent immune checkpoint in those patients with low expression of other well-defined immune checkpoints.

### HHLA2 and CD8 Are Positively Correlated With the Prognosis of Patients With KIRC

**FIGURE 6:**
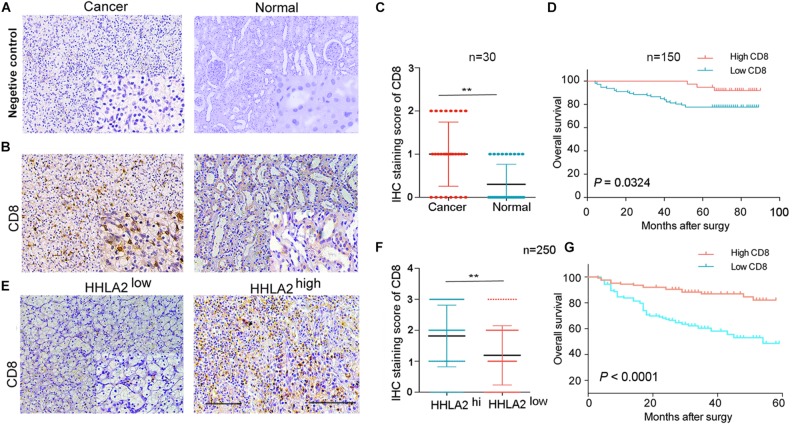
HHLA2 and CD8 are positively correlated with the prognosis of patients with KIRC. **(A)** IHC staining negative control. **(B)** Tumors from a cohort of patients with KIRC (*n* = 180) were stained for CD8 expression at the protein level. **(C)** IHC score of CD8 in normal and tumor tissues form patients with KIRC (paired, *n* = 30). **(D)** High CD8 expression is correlated with a good prognosis (*n* = 150). **(E)** CD8 showed high expression in tumor tissues with high HHLA2 expression. **(F)** IHC score of HHLA2 in tumor tissues form HHLA2-high and low patient groups (*n* = 250). **(G)** High CD8 expression is correlated with a good prognosis (*n* = 250). **P < 0.01 (two-tailed Student’s *t*-test).

Next, we investigated why HHLA2 overexpression in the KIRC tumor sites is correlated with good prognosis. Accumulating evidence has demonstrated that high levels of CD8^+^ T cells predict good prognosis for cancer patients (McKinney et al., 2010). Thus, to assess HHLA2 correlation with CD8, we analyzed CD8 and HHLA2 expression in TMAs of discovery and validation cohorts, with IgG as the negative control (Figure 6A). We found that CD8 showed significantly higher expression in tumor sites than in the matched normal samples (Figure 6B). This pattern was validated in the discovery cohort (Figure 6C, *n* = 30, *P* < 0.01). Furthermore, we divided the patients into two groups according to CD8 expression and found that CD8 levels were positively correlated with the survival time (Figure 6D, *n* = 150, *P* < 0.01). In validation cohort (*n* = 250), we divided the patients into two groups based on HHLA2 levels and subsequently analyzed CD8 expression. CD8 levels were significantly upregulated in tumor tissues of the HHLA2-high group compared with the HHLA2-low group (Figures 6E,F, *n* = 250, *P* < 0.01). A similar trend was observed in the validation cohort (Figure 6G, *P* < 0.0001). Together, these findings suggested that HHLA2 expression is not associated with CD8, while CD8 expression predicts a good prognosis in KIRC.

## Discussion

In the present study, we reported here that HHLA2 can be potentially utilized as a new target for immunotherapy of KIRC. We demonstrated that HHLA2 is widely expressed in a variety of solid tumors, including KIRC, READ, BRCA, and COAD among others. HHLA2 exhibited significantly higher expression in KIRC tumor tissues than in matched normal samples. This abnormal expression may be induced by CNAs or DNA methylation. In tumor sites, HHLA2 expression is positively correlated with survival time. Subsequently, we found that HHLA2 is positively correlated with immune-related activities, but its expression is independent of other B7 and CD28 family members. Finally, by employing clinical samples of patients with KRC, we demonstrated that HHLA2 is positively correlated with CD8 and predicts a good prognosis. These findings indicate the prognostic value of HHLA2 in KIRC and highlight it as a potential target in those patients with limited PD-1 or other well-defined immune checkpoint expression.

HHLA2 belongs to the B7 family and conveys coinhibitory or costimulatory signals through binding to its receptors (Janakiram et al., 2015b). Here, we employed TCGA database and TMAs to investigate the expression pattern of HHLA2 in a several cancer types. Our results showed that HHLA2 was widely expressed in multiple tumor types and HHLA2 protein expression was detected in 12 commonly diagnosed tumors, including breast cancer, lung cancer, and others, several cancers such as colon and rectum cancer exhibited HHLA2 levels in tumor sites, which is in contrast to a previous study (Janakiram et al., 2015a). This discrepancy in expression patterns of HHLA2 in tumors may be explained by the different cohorts and the lack of positive correlation with the B7 family. In our study, patients with KIRC showed higher levels of HHLA2 in cancer tissues than in normal kidney tissue, based on TCGA data. Further analysis showed a positive correlation between higher HHLA2 expression and improved OS. In addition, we extended our analysis to TMAs from 90 patients with KIRC. Consistent with the data from the TCGA dataset, the results demonstrated that high HHLA2 levels in tumors were correlated with a better outcome, supporting the prognostic value of HHLA2 in KIRC.

Specific mechanisms, such as CNAs and DNA methylation, are known to regulate the expression of certain immune-related genes (Diaz-Lagares et al., 2016; Chen G. et al., 2019). Here, we found a significant difference in the proportion of CNAs between HHLA2-high and low groups, suggesting that CNAs may contribute to HHLA2 dysfunction. Indeed, the important role of CNAs in immune responses has been previously reported (Miao et al., 2018). Additionally, we observed that increased DNA methylation showed remarkedly correlation with abnormal HHLA2 expression; similarly, a role for DNA methylation in regulating immune response was also previously reported (Aznar et al., 2018). We further revealed that HHLA2 is positively correlated with immune-related responses, such as IFN-γ production and cell chemotaxis. Indeed, the increased expression of HHLA2 on monocytes, macrophages and B cells after stimulation with lipopolysaccharide and IFN-γ has been described (Janakiram et al., 2015b).

Cancer immunotherapy has entered into a new phase since the discovery of drugs that can interfere with specific immune checkpoints. One of the most reported is the checkpoint proteins are PD-1 and its ligand, PD-L1. However, the clinical efficacy of PD-1/PD-L1 monoclonal antibodies relies on their target expression in the tumor microenvironment, exhibiting limited responses in patients with low PD-1/PD-L1 levels (Herbst et al., 2016; Akinleye and Rasool, 2019). Thus, exploring new immune checkpoint targets is important. Herein, we found that HHLA2 expression is not co-expressed with other B7 or CD28 family members, suggesting that there are numerous factors involved in immune checkpoint expression. Considering the limited correlation of HHLA2 with other immune checkpoints, HHLA2 may represent a good immunotherapeutic target for patients with low levels of PD-L1 or other well-studied checkpoints.

Several studies have reported that HHLA2 is a negative indicator in colon, lung and pancreatic cancers (Cheng et al., 2017; Zhu and Dong, 2018; Yan et al., 2019). The poor prognostic value of HHLA2 was also reported in clear cell RCC (Chen D. et al., 2019; Chen L. et al., 2019). However, increased HHLA2 expression was associated with better post-surgical prognosis in pancreatic and ampullary cancers when using a different anti-HHLA2 antibody clone for IHC (Boor et al., 2020). Herein, we also observed that HHLA2 is a positive predictor in KIRC. Patients with high HHLA2 showed longer survival rates, contradicting previous studies. In KIRC samples, we observed accumulated CD8^+^ T cells in KIRC samples, which secrete IFN-γ. The inducible expression of HHLA2 by cytokines (IFN-γ) has been previously reported (Janakiram et al., 2015b), suggesting that the accumulated effector CD8^+^ T cells can elevate HHLA2 levels. Additionally, HHLA2 was reported to stimulate T cells by interacting with its receptor TMIGD2 (Zhu et al., 2013). Thus, this positive loop between HHLA2 and IFN-γ-secreting CD8^+^ T cells may contribute to its good prognosis in KIRC. Nevertheless, HHLA2 expression on tumor cells has also been reported to stimulate tumor angiogenesis through interactions with TMIGD2 on the endothelium, which may result in a poor prognosis (Janakiram et al., 2015b). These opposing functions highlight that the expression and distribution of HHLA2 and its receptors may determine the reactions and immune responses in tumor microenvironment, further affecting the prognosis. The different datasets or cohorts used in these studies may also lead to the contradictory prognostic value of HHLA2 in cancers. A similar phenomenon was observed for PD-L1 expression and its prognostic significance in various tumors. In colorectal, breast, and ovary cancers, PD-L1 expression represents a better outcome (Darb-Esfahani et al., 2016; Li et al., 2016; Kitano et al., 2017), whereas worse prognostic outcomes were observed in gastrointestinal, esophageal, and pancreatic cancers as well as glioma and hepatocellular carcinoma (Ohaegbulam et al., 2015; Wang et al., 2016; Liu et al., 2017). This highlights that the prognostic value and clinical significance of HHLA2 expression in various cancers may be complex and affected by many factors. It has also been reported that HHLA2 protein is constitutively expressed on the surface of human monocytes and is induced on B cells (Zhao et al., 2013; Janakiram et al., 2015a). However, the HHLA2 expression in B cells or monocytes was not evaluated in our study. Whether the HHLA2 derived from host cells has an impact on patient survival remains unclear. Therefore, further research is necessary to determine the impacts on patient survival of HHLA2 expression derived from host and malignant cells.

## Conclusion

To our knowledge, this is the first work to explore the different prognostic impacts of HHLA2 in solid tumors. We also explored the mechanisms underlying the up-regulation of HHLA2, which predicts a favorable outcome for KIRC. Based on the results, we can speculate that high expression of HHLA2 in KIRC may accompany improved renal function. Furthermore, the positive impact of HHLA2 in KIRC may be explained by a compensatory up-regulation of this molecule in tumor microenvironment, which fights the tumor via an activated immune response. Taking these findings into consideration, immunotherapy based on HHLA2 expression may further improve the prognosis of patients with high HHLA2 expression.

Our study has several limitations. The exact mechanism underlying HHLA2 expression dysfunction was not well-illustrated, which warrants further research. Moreover, there was a limited investigation of HHLA2 receptors as we mainly focused on HHLA2 itself. Nevertheless, this study is the first to show that HHLA2, a newly discovered immune checkpoint ligand, is highly expressed in KIRC and predicts a good prognosis, suggesting it as a potential therapeutic target for patients with KIRC in the clinic.

## Data Availability Statement

Publicly available datasets were analyzed in this study. This data can be found here: https://genomecancer.ucsc.edu/.

## Ethics Statement

The studies involving human participants were reviewed and approved by Ethics Committee of First Affiliated Hospital of Zhengzhou. The patients/participants provided their written informed consent to participate in this study.

## Author Contributions

YZ and XZ conceived and designed this project. ZZ and JL performed experiments and acquired data. ZZ, JL, CZ, FL, LL, DW, DC, and FG analyzed data. All authors participated in writing or revising the manuscript.

## Conflict of Interest

XZ is an inventor on patent number US10093737 (HHLA2 as a novel inhibitor of human immune system and uses thereof) and patent number US 10280208 (TMIGD2 and its derivatives as blockers or binders of cancer-expressed HHLA2 for immunotherapies). The remaining authors declare that the research was conducted in the absence of any commercial or financial relationships that could be construed as a potential conflict of interest.

## Footnotes

1 https://genome-cancer.ucsc.edu/

2 https://www.ncbi.nlm.nih.gov/geo/info/datasets.html

3 https://www.r-project.org

4 http://mexpress.be

5 http://david.ncifcrf.gov/

6 http://amigo.geneontology.org/amigo/landing

## Abbreviations

BLCA: bladder urothelial carcinoma
BRCA: breast invasive carcinoma
CESC: cervical squamous cell carcinoma and endocervical adenocarcinoma
COAD: colon adenocarcinoma
CNAs: copy number variations
CTLA-4: cytotoxic T-lymphocyte -associated protein 4
ESCA: esophageal carcinoma
GBM: glioblastoma multiforme
GO: gene ontology
HNSC: head and neck squamous cell carcinoma
IHC: immunohistochemistry
KICH: kidney chromophobe
KIRC: kidney renal cell carcinoma
KIRP: kidney papillary cell carcinoma
LGG: brain lower grade glioma
LIHC: liver hepatocellular carcinoma
LUAD: lung adenocarcinoma
LUSC: lung squamous cell carcinoma
MHC: the major histocompatibility complex
OV: ovarian serous cystadenocarcinoma
PAAD: pancreatic adenocarcinoma
PCPG: pheochromocytoma and paraganglioma
PD-1: programmed cell death 1
PRAD: prostate adenocarcinoma
RCC: renal cell carcinoma
READ: rectum adenocarcinoma
SARC: sarcoma
SKCM: skin cutaneous melanoma
TCGA: The Cancer Genome Atlas
THYM: thymoma.
